# Angiogenic Capacity of Periodontal Ligament Stem Cells Pretreated with Deferoxamine and/or Fibroblast Growth Factor-2

**DOI:** 10.1371/journal.pone.0167807

**Published:** 2016-12-09

**Authors:** Jessica Ratajczak, Petra Hilkens, Pascal Gervois, Esther Wolfs, Reinhilde Jacobs, Ivo Lambrichts, Annelies Bronckaers

**Affiliations:** 1 Morphology Research Group, Biomedical Research Institute, Hasselt University, Diepenbeek, Belgium; 2 Oral Imaging Center, OMFS-IMPATH Research Group, Department of Imaging and Pathology, Faculty of Medicine, University of Leuven, Leuven, Belgium; Centro Cardiologico Monzino, ITALY

## Abstract

Periodontal ligament stem cells (PDLSCs) represent a good source of multipotent cells for cell-based therapies in regenerative medicine. The success rate of these treatments is severely dependent on the establishment of adequate vasculature in order to provide oxygen and nutrients to the transplanted cells. Pharmacological preconditioning of stem cells has been proposed as a promising method to augment their therapeutic efficacy. In this study, the aim was to improve the intrinsic angiogenic properties of PDLSCs by *in vitro* pretreatment with deferoxamine (DFX; 100μM), fibroblast growth factor-2 (FGF-2; 10ng/mL) or both substances combined. An antibody array revealed the differential expression of several proteins, including vascular endothelial growth factor (VEGF) and placental growth factor (PlGF). ELISA data confirmed a 1.5 to 1.8-fold increase in VEGF for all tested conditions. Moreover, 48 hours after the removal of DFX, VEGF levels remained elevated (1.8-fold) compared to control conditions. FGF-2 and combination treatment resulted in a 5.4 to 13.1-fold increase in PlGF secretion, whereas DFX treatment had no effect. Furthermore, both PDLSCs as pretreated PDLSCs induced endothelial migration. Despite the significant elevated VEGF levels of pretreated PDLSCs, the induced endothelial migration was not higher by pretreated PDLSCs. We find that the observed induced endothelial cell motility was not dependent on VEGF, since blocking the VEGFR1-3 with Axitinib (0.5nM) did not inhibit endothelial motility towards PDLSCs. Taken together, this study provides evidence that preconditioning with DFX and/or FGF-2 significantly improves the angiogenic secretome of PDLSCs, in particular VEGF and PlGF secretion. However, our data suggest that VEGF is not the only player when it comes to influencing endothelial behavior by the PDLSCs.

## Introduction

A decade ago, a mesenchymal stem cell (MSC)-like cell population was also discovered in the periodontal ligament of human teeth [1]. These periodontal ligament stem cells (PDLSCs) have been identified as a good source of multipotent cells for cell-based therapies in regenerative medicine. However, a major concern is the survival of these stem cells after transplantation. Injured tissue is usually poorly perfused resulting in a lack of oxygen and nutrients for both grafted and resident cells [2]. It has only recently been demonstrated that PDLSCs possess the ability to stimulate angiogenesis [3]. Furthermore, Yeasmin et al. showed that PDLSCs secrete soluble pro-angiogenic factors such as vascular endothelial growth factor (VEGF) and fibroblast growth factor-2 (FGF-2) and induce blood vessel formation after co-transplantation with endothelial cells (EC)[3].

In light of the recent insight that the paracrine actions of MSCs are responsible for their tremendous therapeutic potential, researchers have been investigating different approaches to modulate and enhance the MSC-secretome [4]. Hypoxia is a potent stimulus for the secretion of numerous trophic factors. Not surprisingly, hypoxic preconditioning has gained a lot of attention as a method to improve the paracrine actions of a variety of stem cell sources [2]. Various research groups have already indicated that low oxygen levels, increase stem cell survival and the secretion of VEGF and FGF-2. Moreover, as a consequence, this method also led to increased angiogenesis in an *in vivo* model of murine hind limb ischemia [5–8]. Despite the proven success of hypoxic preconditioning, mimicking hypoxia using pharmacological pretreatment could represent a more convenient alternative [2]. Deferoxamine (DFX) is such a chemical agent which simulates hypoxia by inhibiting the activity of prolyl hydroxylase (PHD), a key enzyme of the oxygen sensing pathway. [9]. This drug is an FDA-approved iron chelator used to treat iron overload diseases and has been reported to increase VEGF secretion of both dental pulp-derived cells and cells derived from the periodontal ligament [10, 11]. Besides hypoxia mimicking agents there is a plethora of cytokines, growth factors and chemical agents that have been investigated for their potential to augment the angiogenic profile of stem cells. Pretreatments such as IL-1α [12] and TNF-α [13] have been described to increase VEGF secretion in PDLSC and adiponectin stimulates PDLSC proliferation and wound healing [14]. In the present study, we aimed to improve the angiogenic capacities of PDLSCs by *in vitro* preconditioning with DFX, FGF-2 or a combination of both substances.

## Materials and Methods

### Cell culture

Periodontal ligaments were obtained from patients (16–27 years of age) undergoing extraction of third molars for orthodontic or therapeutic reasons at Ziekenhuis Oost Limburg, Genk, Belgium. All participants provided written informed consent, in the case of patients under the age of 18, informed consent was obtained via their guardians. This study protocol and consent procedure was approved by the medical ethical committee from Hasselt University. Periodontal ligaments were removed from extracted molars with forceps and rinsed with Minimal Essential Medium, alpha modification (αMEM, Sigma-Aldrich, St. Louis) supplemented with 100 U/mL Penicillin and 100 μg/mL Streptomycin (Sigma), 2 mM L-glutamine (Sigma) containing 10% FBS (BiochromAG, Berlin, Germany), after which periodontal ligament stem cells (PDLSCs) were isolated according to the explant method as described in detail previously [15].

A human microvascular endothelial cell line (HMEC-1; [16]) was purchased from the Center of Disease Control and Prevention (Atlanta, GA, USA). HMEC-1 were cultured in MCDB 131 medium (Invitrogen, Carlsbad, CA) as previously described [15].

### PDLSC differentiation

Adipogenic, osteogenic and chondrogenic differentiation of PDLSCs was induced as previously described [17]. Briefly, PDLSCs were cultured in adipogenic induction medium (StemXVivo^™^; R&D Systems, UK; CCM011) containing hydrocortisone, isobutylmethylxanthine and indomethacin. After 3 weeks adipogenic differentiation was evaluated based on the presence of lipid droplets, which were identified using 0.3% Oil Red O staining. Osteogenic differentiation was induced using medium (StemXVivoTM; R&D Systems, UK; CM008) supplemented with dexamethasone, ascorbatphosphate and β-glycerolphosphate. Osteogenic differentiation was assessed by the production of extracellular matrix and calcium deposits, which were visualized using Alizarin Red S staining. Differentiation of PDLSCs to chondrogenic cells was performed according to manufacturer’s guidelines (R&D systems). A pellet containing 250 000 PDLSCs was subjected to chondrogenic differentiation medium consisting of 1% insulin–transferrin–selenious acid (ITS) supplement and 1% chondrogenic supplement (R&D systems). This supplement contained dexamethasone, ascorbate-phosphate, proline, pyruvate and transforming growth factor-β3. Chondrogenic differentiation was verified by staining for aggrecan, in order to assess the presence of cartilagenous extracellular matrix.

### Preconditioning of PDLSCs

**Fig 1:**
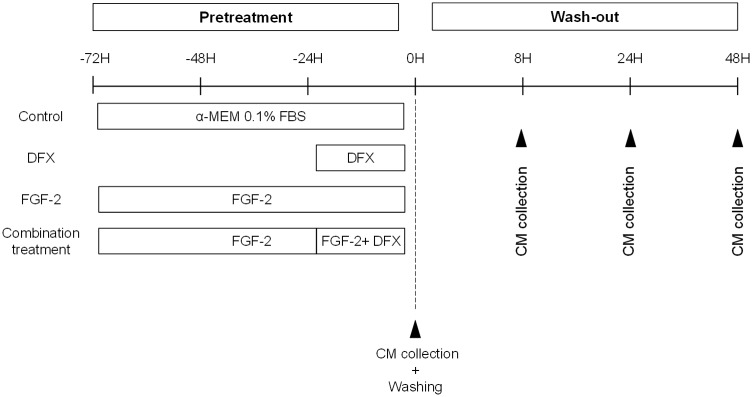
Schematic overview of PDLSC pretreatment procedures. After overnight incubation standard PDLSCs culture medium was switched to standard medium containing only 0.1% FBS, supplemented with either 100μM DFX or 10 ng/mL FGF-2. CM of DFX-treated cells was collected after 24 hours whereas CM of FGF-2-treated cells was collected after 72 hours. In the case of combination treatment, DFX was added 48 hours after the start of FGF-2 pretreatment and CM was collected 24 hours later. Subsequently cells were washed and priming media were replaced by standard culture medium containing 0.1% FBS. This wash-out medium was collected after 8, 24 and 48 hours.

With regard to pharmacological pretreatment, PDLSCs were treated with DFX (Calbiochem, Milipore Corp., Billerica, MA, USA), FGF-2 (Immunotools, Friesoythe, Germany) or both substances combined (Fig 1). PDLSCs (passages 3–8) were seeded in a 6-well plate at a density of 10 405 cells/cm^2^ in standard PDLSC culture medium. The next day cells were rinsed with PBS and standard culture medium was replaced by 1.4 mL standard medium containing only 0.1% FBS either supplemented with 100 μM DFX or 10 ng/mL FGF-2. Conditioned medium (CM) containing the PDLSC-secretome of DFX-treated cells was harvested after 24 hours, while CM of FGF-2-treated PDLSCs was harvested after 72 hours. For the combined treatment, PDLSCs were first exposed to FGF-2 and after 48 hours 100 μM of DFX was added to the medium for an additional 24 hours before CM was harvested. To evaluate the production of vascular endothelial growth factor (VEGF) and placental growth factor (PlGF) after pharmacological removal, a wash-out (WO) experiment was performed. Therefore, cells were rinsed with PBS and priming medium was replaced with standard PDLSC culture medium containing 0.1% FBS. To evaluate how the VEGF production was affected in time after removal of the priming agent, wash-out CM was harvested at 8, 24 and 48 hours. All CM was stored at -80°C until further use.

### Flow cytometric analysis

The expression of stem cell surface markers was evaluated with flow cytometry before and after priming. PDLSCs were tested for the lack of hematopoietic cell surface markers: CD34 and CD45 and the expression of MSC surface markers: CD44, CD73, CD90, CD105. S1 Table summarizes the used detection antibodies and matched isotype controls. Flow cytometric analysis was performed under standard culture conditions (10% FBS), under low serum conditions (0.1% FBS) and after pretreatment.

After priming, PDLSCs were harvested by trypsinization and resuspended in PBS containing 2% FBS and cells were incubated for 30 minutes at room temperature to allow re-expression of cellular markers. Next, cells were incubated with fluorescently labeled antibodies for 45 minutes. Matched isotype controls were included as a negative control for nonspecific background staining. After incubation, PDLSCs were washed three times followed by fluorescent activated flow cytometry analysis on a FACS Calibur flow cytometer equipped with CellQuest Pro software (BD Biosciences, Franklin Lakes, N.J., USA). For each condition 10,000 cells were read out.

### Antibody array

In order to analyze the angiogenesis-related proteins secreted by treated and untreated PDLSCs, the human angiogenesis array kit (R&D systems) was used as a general screening tool. This array was performed according to the manufacturer's instructions. Conditioned medium of treated and untreated PDLSCs was incubated with a detection antibody cocktail for one hour, prior to adding this mixture to the array membranes. Membranes were incubated overnight and after rinsing, streptavidin-HRP was added and incubated for 30 minutes, at room temperature. Immunoreaction was visualized with chemiluminescent reagents (ECL plus, GE healthcare, Little Chalfont, UK). Finally the membranes were exposed to X-ray films for different time points and the results were analyzed by ImageJ using a Dot Blot analyzer plug-in.

### Enzyme-linked immunosorbent assay

To evaluate the concentration range of the produced angiogenic factors VEGF and PlGF an enzyme-linked immunosorbent assay (ELISA) was performed on CM of PDLSCs after DFX, FGF-2 and combination treatment. ELISA’s for VEGF (Raybiotech, USA) and PlGF (Boster, USA) were performed according to manufacturer’s instructions and at least five different patient samples were tested.

### 3-(4,5-Dimethylthiazol-2-yl)-2,5-diphenyltetrazolium bromide assay

In order to evaluate the effect of PDLSCs on the proliferation of endothelial cells a 3-(4,5-Dimethylthiazol-2-yl)-2,5-diphenyltetrazolium bromide (MTT)–assay was performed. HMEC-1 (passages 3–9) were seeded at 31 250 cells/cm^2^ in a flat bottom 96-well plate. After overnight incubation, HMEC-1 were washed with PBS and incubated for 48 hours with either CM of PDLSCs, standard PDLSC culture medium containing 0.1% FBS (negative control) or standard PDLSC culture medium containing 10% FBS (positive control). An additional negative control for FGF-2 and combination treatment was added, containing the priming agents, in order to correct for the presence of FGF-2 and DFX in the CM. All conditions were performed in triplicate. After 48 hours of culturing, media were replaced with 500 μg/mL MTT in standard PDLSC culture medium containing 0.1% FBS. Four hours later, the MTT solution was removed and replaced with a DMSO (Sigma) and 0.01 M Glycine (Sigma) mixture to dissolve formazan crystals. Absorbance was measured with a Benchmark microplate reader (Bio-Rad Laboratories, Hercules, CA) at a wavelength of 570 nm. This assay was performed on a total of eight patient samples.

### Transwell migration assay

HMEC-1 migration towards PDLSC CM of treated and untreated PDLSCs was measured with a transwell migration assay, using a 24-well plate with inserts containing a filter with an 8 μm pore size (Thincert^™^, Greiner Bio-One). For migration towards treated and untreated cells, PDLSCs (passage 4–5) were seeded in a 24-well plate at a density of 25 000 PDLSCs/cm^2^ and left for culturing overnight. The next day, PDLSCs were rinsed with PBS and pretreatment was started. For the migration of HMEC-1 towards CM, CM of treated and untreated PDLSCs was added to the wells before adding the culture inserts. Inserts were seeded with 100 000 HMEC-1 in standard PDLSC culture medium with 0.1% FBS and placed over the wells. Standard PDLSC culture medium with either 0.1% FBS or 10% FBS were added to the wells underneath as negative and positive controls respectively. Furthermore, media containing 10 ng/mL FGF-2 or 10 ng/mL FGF-2 and 100 μM DFX were used as controls for FGF-2 and combination treatment. In order to eliminate the effect of VEGF on endothelial migration, VEGFR1-3 on HMEC-1 were blocked by adding 0.5 nM Axitinib (Pfizer, New York, USA) to the culture inserts (n = 4). After 24 hours of transmigration, HMEC-1 cells were fixed with 4% paraformaldehyde (PFA) for 20 minutes at room temperature and stained for analysis with 0.1% crystal violet in 10% ethanol for 10 minutes at room temperature. Two 10x representative pictures were taken per insert with an inverted phase-contrast microscope (Nikon, Eclipse TS100) equipped with a ProgRes^®^ C3 digital microscope camera (Jenoptik AG, Jena, Germany). The degree of migration was expressed as mean area covered with cells (in percentage) and quantified with AxioVision software 4.6.3 (Carl Zeiss Vision, Aalen, Germany). Migration assays were independently performed on at least eight different patient samples.

### Chorioallantoic membrane assay

Angiogenic properties of treated and untreated PDLSCs were tested *in vivo* with a chorioallantoic membrane assay (CAM). Fertilized white leghorn chicken eggs (*Gallus gallus*) were incubated in a humidified atmosphere at 37°C for three days. At 3 days of embryonic age (E3), albumin was removed to detach the developing CAM from the eggshell. At E9, eggs with exposed CAM were incubated with plastic discs containing 500 ng recombinant human VEGF 165 (R&D systems) or 50,000 pretreated or untreated PDLSCs dissolved in growth factor-reduced Matrigel^™^ droplets (BD Biosciences). Plastic discs containing pure Matrigel^™^ droplets were used as a negative control. Eggs were incubated for three consecutive days and at E12 the CAM was dissected out of the eggs to analyze angiogenesis. Images were taken with a Sony HDR-XR350VE handycam camera (Sony corporation, Tokyo, Japan) and quantified by drawing a circle (radius 4 mm) over the plastic discs on the picture and counting intersecting blood vessels. Blood vessels were counted by two different researchers in a blinded fashion.

### Statistical analysis

Statistical analysis was performed using Graphpad Prism software 5.03 (Graphpad Software, La Jolla, CA). Data normality was tested with D’Agostino & Pearson normality test. When Gaussian distribution was reached, experimental groups were compared using a one-way analysis of variance (ANOVA) with a Bonferroni post-test for groups ≤ 5. Non-parametric data were evaluated with a Kruskall-Wallis test combined with Dunn’s post-test. ELISA data were statistically analyzed by means of a two-way ANOVA with a Bonferroni post-test for multiple comparisons. Statistical significance was reached at p-values ≤ 0.05. All data were expressed as mean ± standard error of the mean (SEM).

## Results

**Fig 2:**
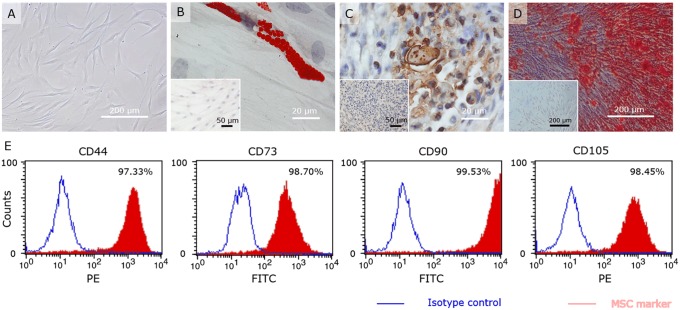
Characterization of human periodontal ligament stem cells. PDLSCs display mesenchymal-like cell morphology comprising spindle-shaped and polygonal cell types (A). Successful adipogenic, chondrogenic and osteogenic induction (n = 3) is confirmed by histochemical staining of respectively lipid droplets with Oil red O (B), aggrecan (C) and the presence of calcified matrix by means of Alizarin Red S staining (D). Furthermore, flow cytometric-analysis showed the expression of MSC-markers CD44, CD73, CD90 and CD105 (E).

The resulting PDLSC population displayed mesenchymal-like cell morphology comprising spindle-shaped and polygonal cell types (Fig 2A). The immunophenotype was evaluated by means of flow cytometry, which showed a high expression level of the stem cell markers: CD44, CD73, CD90 and CD105 (Fig 2E). In order to assess the multilineage differentiation potential, PDLSCs were differentiated towards adipogenic, chondrogenic and osteogenic lineages (Fig 2B–2D). Successful adipogenic, chondrogenic and osteogenic induction was confirmed by histochemical staining of respectively lipid droplets with Oil red O, aggrecan a major structural protein of cartilage and staining of calcified matrix by means of Alizarin Red S staining. Together these data demonstrate that the PDLSC populations used in this study comply with the minimal requirements for mesenchymal stem cells [18].

### Effect of DFX and FGF-2 on the angiogenic profile of PDLSC

**Fig 3:**
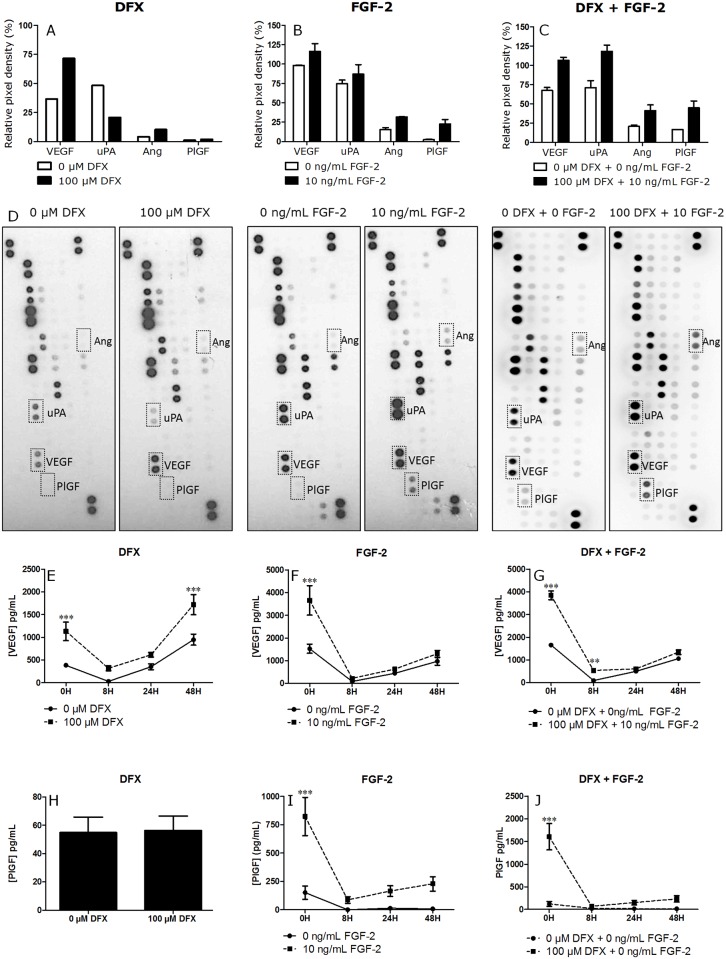
Treatment with DFX and/or FGF-2 increases VEGF and PlGF secretion of PDSLCs. PDLSCs were pretreated either with 100 μM DFX for 24 hours, with 10 ng/mL FGF-2 for 72 hours or with a combination of both substances. (A-D) Antibody array of the CM of PDLSCs pretreated with DFX (n = 1), FGF-2 and the combination treatment (n = 2). Graphs A-C show pixel density of the differentially expressed proteins. As determined by ELISA (n = 5), DFX treatment resulted in a 1.8 fold increase in VEGF secretion (E), whereas FGF-2 treatment increased VEGF secretion 1.5 fold (F). Finally, a combination of both agents led to a 2.7 fold increase in VEGF secretion (G). ELISA of PlGF showed that not DFX (H) but treatment with FGF-2 (I) and FGF-2 combined with DFX (J) significantly upregulated PlGF secretion. Data are expressed as mean ± SEM and analyzed with Two-Way-ANOVA, ** = p ≤ 0.01; *** = p ≤ 0.001.

The first part of this study focused on the analysis angiogenic factors secreted by PDLSCs before and after pretreatment. PDLSCs were either treated with 100 μM DFX for 24 hours, 10 ng/mL FGF-2 for 72 hours or with a combination of both substances. Dot blot analysis of CM of untreated and pretreated PDLSCs was used to investigate the relative expression of a wide variety of angiogenic proteins secreted by PDLSCs (Fig 3A–3D). Dot blot analysis revealed an increase in VEGF secretion after pretreatment as well as a differential secretion of other angiogenic factors such as angiogenin (Ang), urokinase (uPA), and placental growth factor (PlGF). As the difference in secretion of VEGF and PlGF was the most pronounced, the production of both factors was validated by means of ELISA (Fig 3E–3J).

The production of VEGF of untreated and pretreated PDLSCs was determined by means of ELISA (Fig 3E–3G). Pretreating PDLSCs with 100 μM DFX for 24 hours resulted in a 1.8 fold increase in VEGF and pretreatment with 10 ng/mL FGF-2 for 72 hours resulted in an 1.5 fold increase compared to untreated PDLSCs. When both factors were combined VEGF secretion increased 2.7 fold. For combination therapy, first 10 ng/mL FGF-2 was added to the culture medium and 48 hours later, the medium was supplemented with 100 μM DFX. The CM was harvested after a total incubation of 72 hours. Furthermore, the effect of DFX persisted, even when it was removed from the culture medium (wash-out) VEGF secretion remained elevated, 1.8 fold (Fig 3E).

When cells were treated with FGF-2 no difference in VEGF levels was present after withdrawal of the growth factor (Fig 3F). Following combination treatment however, the effects persisted for 8 hours, with no further difference between the VEGF secretion of pretreated and untreated PDLSCs afterwards. PlGF secretion increased significantly after FGF-2 and combination treatment, 5.4 fold and 13.1 fold respectively (Fig 3I–3J). Whereas DFX treatment did not influence PlGF secretion (Fig 3H). After the removal of FGF-2 or FGF-2 and DFX in the case of combination treatment, the levels of PlGF remained elevated, 25.5 fold and 14.4 fold respectively, in the treated PDLSCs compared to untreated PDLSCs, however no significance could be stated (Fig 3I–3J).

**Fig 4:**
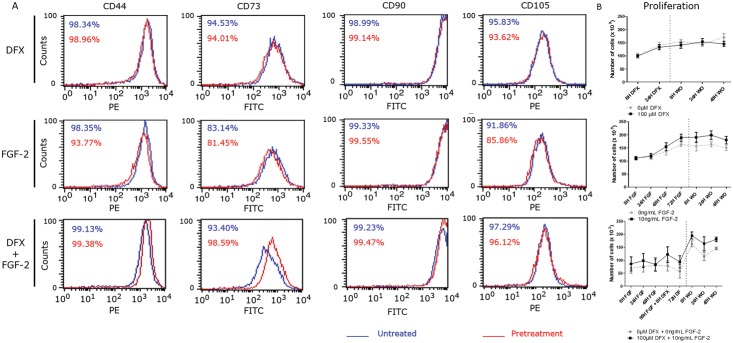
The effect of DFX, FGF-2 or combination treatment of the MSC marker expression as demonstrated by flow cytometry or on cell proliferation (measured with Trypan Blue Exclusion assay). (A) Pretreatment does not influence MSC marker expression of PDLSC proliferation (n = 6). (B) Cell proliferation was monitored during treatment by means of Trypan Blue Exclusion assay (n = 5).

With flow cytometry the effect of the FGF-2 and DFX pretreatment on the expression of stem cells markers was analyzed. Preconditioning did not influence PDLSC surface marker expression or proliferation rates (Fig 4).

### PDLSC induce endothelial migration *in vitro*

**Fig 5:**
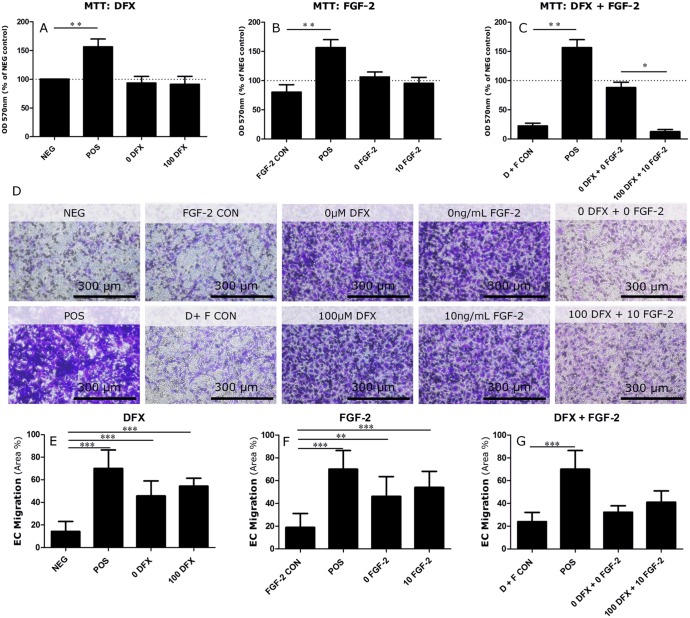
Effects of control and primed periodontal ligament stem cells on endothelial cell behavior *in vitro*. (A) CM of control PDLSCs or cells treated with DFX or FGF-2 alone does not induce HMEC-1 proliferation as determined by MTT (n = 8). (B) PDLSCs induce endothelial cell migration in the transwell migration assay. Both untreated and treated PDLSCs are able to induce HMEC-1 migration, but there is no difference in chemotactic potential between control and primed PDLSCs. Negative control: culture medium containing 0.1% FBS. Positive control: culture medium containing 10% FBS. FGF control: culture medium conta0ining 10 ng/mL FGF-2. D+F control: culture medium containing 100 μM DFX and 10 ng/mL FGF-2. Scale bar = 300 μm. Data are expressed as mean ± SEM. ** = p-value ≤0.01; *** = p-value ≤ 0.001 compared to either FGF-2 control or D+F control.

**Fig 6:**
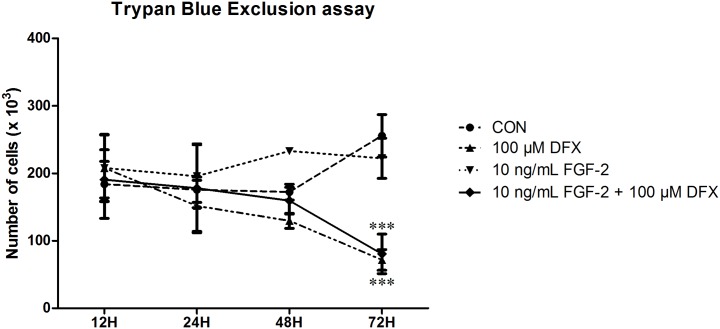
Exposure to DFX leads to decreased cell numbers of endothelial cells after 72 hours. Cell numbers were monitored via Trypan Blue Exclusion, only 72 hours of DFX treatment resulted in a decreased cell count (n = 3). Control: culture medium containing 0.1% FBS. The conditions included in this experiment only contained the different priming agents and were lacking factors secreted by PDLSCs. DFX = deferoxamine; FGF-2 = fibroblast growth factor-2; PDLSC = periodontal ligament stem cells. Data are expressed as mean ± SEM and were analyzed by Two-Way ANOVA. *** = p-value ≤0.001.

As angiogenesis is a complex multistep process, it is not only important to determine the factors secreted by PDLSCs but it is also crucial to assess their influence on endothelial cell behavior. Therefore, several *in vitro* assays were performed in order to mimic the different stages of angiogenesis. One of the first steps is the proliferation of endothelial cells. The capacity of PDLSCs to influence endothelial cell metabolism was investigated by means of an MTT assay (Fig 5A–5C). For this purpose HMEC-1 were cultivated in CM of either untreated or DFX or FGF-2 treated PDLSCs for 48 hours after which no difference in metabolic activity could be detected. However, CM harvested from PDLSCs receiving combination treatment seemed to significantly decrease HMEC-1 viability. This effect can be ascribed to an interaction between DFX and MTT. To further demonstrate that the decrease in viability is due to technical difficulties and not to the presence of DFX itself, cell numbers were monitored with the Trypan Blue Exclusion assay. Only after being exposed to 100 μM DFX for 72 hours, cell numbers started to decline (Fig 6).

During angiogenesis, endothelial cells also respond to chemotactic stimuli, which direct them to the site in need of vascularization. Therefore, the chemotactic potential of untreated and treated PDLSCs was evaluated by means of a transwell migration assay. In order to induce transmigration, either treated or untreated PDLSCs were seeded in bottom wells, after overnight incubation, medium was changed to α-MEM 0.1% FBS. Twenty-four hours prior to the priming endpoint, HMEC-1 were added in culture inserts.

Both treated and untreated PDLSCs were capable of inducing transmigration of HMEC-1 (Fig 5D–5G). However, no difference could be stated between treated and untreated PDLSCs. Despite the fact that combination treatment resulted in a 2.7 fold increase in VEGF secretion compared to 1.8 fold for DFX treatment and 1.5 fold for FGF-2 treatment, the effect on endothelial migration was the least pronounced in this condition (Fig 5G). In order to eliminate the possibility that the induced migration was due to the presence of FGF-2 or DFX in the CM, control samples were included containing equal concentrations of FGF-2 or DFX, but lacking growth factors secreted by PDLSCs.

### PDLSCs induce endothelial cell migration independent of VEGF

**Fig 7:**
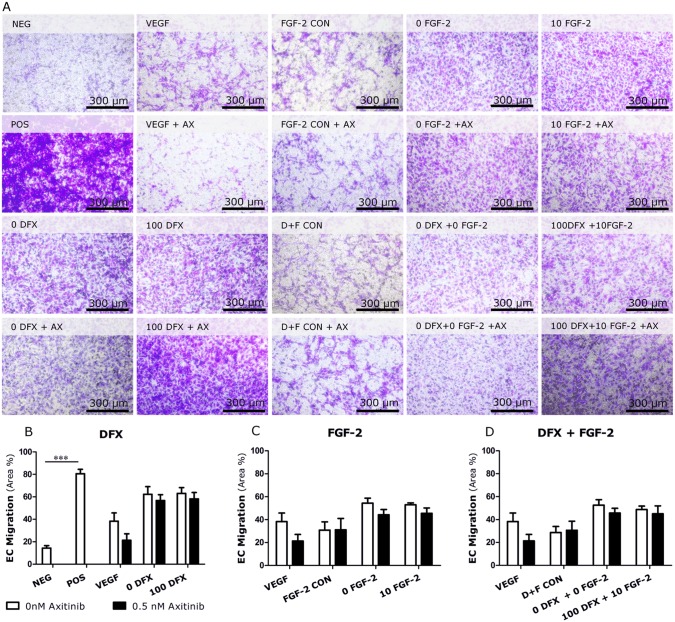
Periodontal ligament stem cells do not elicit endothelial cell migration via VEGF. Conditioned medium of untreated and treated PDLSCs induces endothelial cell migration. In the presence of 0.5 nM Axitinib (AX), a VEGFR1-3 inhibitor, endothelial cells continue to migrate towards factors secreted by PDLSCs. Negative control: culture medium containing 0.1% FBS. Positive control: culture medium containing 10% FBS. FGF control: culture medium containing 10ng/mL FGF-2. D+F control: culture medium containing 100 μM DFX and 10 ng/mL FGF-2. Scale bar = 300μm. Data are expressed as mean ± SEM. ** = p-value ≤0.01; *** = p-value ≤ 0.001 compared to either FGF control or D+F control.

Despite the significant increase in VEGF secretion after pretreatment, pretreated PDLSCs do not cause a greater endothelial cell migration. In order to investigate whether chemotaxis is dependent on VEGF, migration assays were performed with HMEC-1 cells exposed to Axitinib, which is a potent VEGFR1-3 inhibitor (Fig 7) [19]. A first indication that VEGF is not solely responsible for the induction of endothelial migration is the fact that 100 ng/mL of recombinant VEGF causes only a minimal increases in endothelial motility. This migration is inhibited by adding 0.5 nM Axitinib to the culture inserts containing HMEC-1. The presence of Axitinib has little to no impact on the endothelial migration elicited by the CM of either pretreated or untreated PDLSCs. This suggests that this migration is not dependent on VEGF.

### PDLSCs do not induce angiogenesis *in ovo*

**Fig 8:**
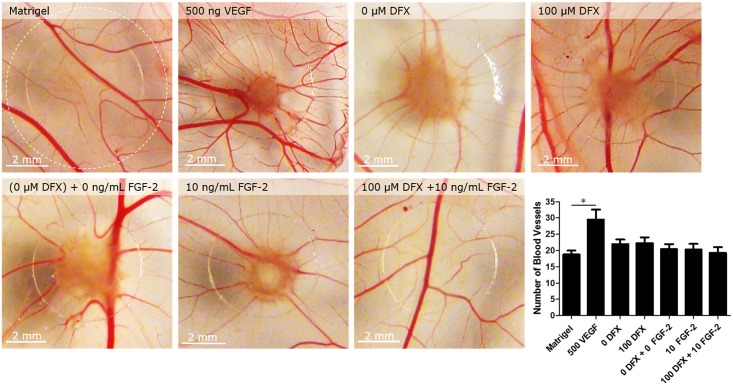
PDLSCs do not induce angiogenesis *in ovo*. Images on the left show representative vascularization of the chorioallantoic membrane following three days of incubation with growth factor reduced Matrigel^™^ containing either 500 ng VEGF (n = 13), untreated PDLSCs cultured in low serum conditions for 24 (n = 27) or 72 hours (n = 26) or PDLSCs treated with 100μM DFX (24h, n = 24), 10ng/mL FGF-2 (72h, n = 24)) or a combination therapy (n = 21). Neither untreated (24h control, 72h control) nor treated PDLSCs were able to increase the average number of blood vessels. Scale bar = 2mm. Data are expressed as mean ± SEM. PDLSCs = periodontal ligament stem cells; VEGF = vascular endothelial growth factor; DFX = deferoxamine; FGF-2 = fibroblast growth factor-2.

In order to investigate whether PDLSCs could induce angiogenesis in an *in vivo* setting, a CAM assay was performed. In this assay, treated and untreated PDLSCs were dissolved in growth factor-reduced Matrigel^™^ and placed onto the developing chorioallantoic membrane of a fertilized chicken egg at E9. Pure Matrigel^™^ droplets were used as a negative control. After three days of incubation, the CAM was removed and pictures were taken. In order to quantify angiogenesis, a concentric circle (radius = 4mm) was drawn and intersecting blood vessels were counted. However, neither untreated nor treated PDLSCs increased the number of blood vessels compared to control conditions (Fig 8). In contrast, incubation with 500 ng of human recombinant VEGF resulted in increased blood vessel formation.

## Discussion

Since supporting vasculature is necessary for the survival of the transplanted cells/tissues, the efficiency of regenerative medicine could be ameliorated by enhancing the angiogenic potential of stem cells [2]. In this study, we investigated the possibility of boosting the angiogenic potential of PDLSCs by means of *in vitro* preconditioning with DFX and/or FGF-2.

PDLSCs were found to express a characteristic set of MSC markers, with very little variation between different populations. High expression levels of these MSC markers together with their adherence to plastic and multilineage differentiation potential, qualify the PDLSCs used in this study as multipotent mesenchymal stromal cells according to the minimal requirements provided by the society for cellular therapy [18].

Pharmacological interventions to increase stem cell survival and biological activities, prior to transplantation, are considered one of the most promising strategies for future applications in regenerative medicine [2]. In order to enhance the angiogenic potential of PDLSCs, cells were primed with FGF-2 or with the iron chelator DFX. The initial screening of the PDLSC secretome revealed a differential secretion of several angiogenesis related factors after pretreatment, including VEGF and PlGF. A number of studies have already implicated VEGF as a major player in the process of angiogenesis [20, 21]. VEGF has been described to regulate vessel permeability and to stimulate endothelial proliferation and migration [22]. PlGF is a member of the VEGF family, and like VEGF, it binds to the VEGF-1 receptor [23]. Array results were validated by means of ELISA, which showed a significant upregulation of VEGF secretion after pretreatment for all three conditions. Moreover, 48 hours after removal of DFX from the culture medium, VEGF levels remained elevated in the pretreated PDLSC populations. This wash-out effect is important with regards to future *in vivo* applications, since it indicates a prolonged effect of the *in vitro* priming with DFX. Similar effects of DFX preconditioning have already been reported with regard to BM-MSCs [24] and adipose-derived stem cells (ASCs) [25]. Moreover, Agis and colleagues already reported an increased VEGF secretion after treating periodontal ligament fibroblasts with DFX [11]. Whereas, Yanagita and colleagues demonstrated an increased VEGF secretion in FGF-2-primed mouse PDL cells. The report mentions similar results were shown using human PDL cells however no data are shown [26]. Combining FGF-2 and DFX priming is to our knowledge a unique and previously undescribed approach.

In order to assess the functional effects of PDLSCs on the different steps of angiogenesis, several *in vitro* tests were performed with HMEC-1. Despite the substantial secretion of VEGF, neither untreated nor pretreated PDLSCs had an effect on the proliferation of HMEC-1. These results are in agreement with the study by Liu et al. who reported no effect of DFX pretreated ASCs on HMEC-1 proliferation. Furthermore, previous reports from our lab also demonstrated no effect of dental pulp stem cells (DPSCs), stem cells from the apical papilla (SCAPs) and of the dental follicle (FSCs) on HMEC-1 proliferation [15]. Surprisingly, MTT results indicated a major decrease in HMEC-1 viability when exposed to CM of PDLSCs receiving combination treatment. This effect can probably be ascribed to an interaction between DFX and MTT, which was also reported by Agis and colleagues [11]. Furthermore, Trypan Blue Exclusion assay confirmed that exposure to 10 ng/mL FGF-2 and 100 μM DFX for 48 hours did not influence HMEC-1 proliferation.

A second important step in the process of blood vessel formation is endothelial migration [27]. Both untreated and pretreated PDLSCs induced EC motility. However, despite the increase in VEGF secretion, pretreatment of PDLSCs did not result in an increase in endothelial migration. In order to evaluate whether the HMEC-1 migration is dependent on VEGF, Axitinib was used to block VEGFR1-3. Both untreated and pretreated PDLSCs were able to induce migration when HMEC-1 were exposed to 0.5 nM Axitinib. We showed that recombinant VEGF is able to attract HMEC-1 and this process can be reversed by adding 0.5 nM Axinitib. Together, these data indicate that the *in vitro* chemotaxis of HMEC-1 towards PDLSCs is not dependent on VEGF alone but is possibly the result of a synergistic effect of several chemotactic factors secreted by both untreated and primed PDLSCs.

Finally, in order to assess the angiogenic potential of untreated and primed PDLSCs *in vivo* a CAM assay was performed. Unfortunately this study was unable to demonstrate an increased blood vessel formation in the presence of PDLSCs. VEGF, as used in this and numerous other studies (27, 28), as a positive control, significantly induced blood vessel formation. Thus despite the induced endothelial cell migration, no increase on *in vivo* angiogenesis was seen. Our lab previously demonstrated the ability of DSPCs and SCAPs [15] to enhance CAM angiogenesis. However, no induction of CAM angiogenesis was reported for the FSC population. This might in part explain the lack of PDLSC-induced angiogenesis, since the dental follicle eventually develops into the periodontal ligament and both populations are therefore closely related [28]. Nevertheless, also the priming by DFX and FGF-2 and thus the induced VEGF and PlGF secretion was not able to compensate the lack of angiogenic activities of PDLSCs *in vivo*.

In conclusion, this study provides evidence that pretreatment of PDLSCs with DFX and FGF-2 is able to upregulate VEGF and PlGF production, and in the case of DFX even after the removal of the priming agent. Especially the presence of a wash-out effect could have major implications in future uses of DFX primed cell transplants. Furthermore, we demonstrated that both untreated as well as pretreated PDLSCs were able to induce endothelial migration, however, this migratory response was not dependent on VEGF secretion. This VEGF independent response may therefore in part explain the lack of functional effects of PDLSCs on endothelial proliferation and blood vessel development in the CAM assay, despite their potent secretome. For future attempts to enhance angiogenic effects of stem cells, it might be important to focus on other inducers of the angiogenic cascade then the extensively-studied VEGF.

## Supporting Information

S1 Table Primary antibodies and matched isotype controls used for flow cytometry. PE: Phycoerythrin; FITC: fluorescin isothiocyanate.
(DOCX)
