# Supplementary material for: Angiogenic Capacity of Periodontal Ligament Stem Cells Pretreated with Deferoxamine and/or Fibroblast Growth Factor-2
Source: PLoS One. 2016 Dec 9;11(12):e0167807. doi: 10.1371/journal.pone.0167807 (PMC5147980; doi:10.1371/journal.pone.0167807)
Supplement: S1 Table — PE: Phycoerythrin; FITC: fluorescin isothiocyanate. (DOCX) [file pone.0167807.s001.docx]

**Table S1**:Primary antibodies and matched isotype controls used for flow cytometry

| Antibody | Species | Supplier | Isotype | Supplier | Dilution |
| --- | --- | --- | --- | --- | --- |
| CD 34 PE | mouse | Immunotools | IgG1 PE | eBioscience | 1:100 |
| CD 45 PE | mouse | eBioscience | IgG1 PE | eBioscience | 1:100 |
| CD 44 PE | mouse | Immunotools | IgG2b PE | eBioscience | 1:100 |
| CD 73 FITC | mouse | eBioscience | IgG1 FITC | eBioscience | 1:100 |
| CD 90 FITC | mouse | eBioscience | IgG1 FITC | eBioscience | 1:100 |
| CD 105 PE | mouse | eBioscience | IgG1 PE | eBioscience | 1:100 |

PE: Phycoerythrin; FITC: fluorescin isothiocyanate
